# Prevalence and associated factors of basilar artery dolichosis in patients with acute cerebral infarction

**DOI:** 10.3389/fmed.2023.832878

**Published:** 2023-02-23

**Authors:** Shugang Cao, Mingfeng Zhai, Jun He, Ping Cui, Tingting Ge, Jian Wang, Wen’an Xu, Rongfeng Wang, Mingwu Xia

**Affiliations:** ^1^Department of Neurology, Hefei Hospital Affiliated to Anhui Medical University, Hefei, China; ^2^Department of Neurology, The People’s Hospital of Fuyang, Fuyang, China; ^3^Department of Radiology, Hefei Hospital Affiliated to Anhui Medical University, Hefei, China

**Keywords:** stroke, cerebral infarction, basilar artery dolichosis, vertebrobasilar dolichoectasia, diabetes mellitus

## Abstract

**Introduction:**

Little attention has been given to the factors associated with basilar artery (BA) dolichosis. This study aims to elucidate the prevalence and associated factors of BA dolichosis in patients with acute cerebral infarction (ACI).

**Methods:**

We collected the clinical and laboratory data of 719 patients with ACI admitted to our department. Magnetic resonance angiography was used to evaluate the geometric parameters of the BA and intracranial vertebral arteries (VAs). A BA curve length > 29.5 mm or bending length (BL) > 10 mm was identified as BA dolichosis. Univariate and multivariate logistic regression were performed to determine the factors associated with BA dolichosis.

**Results:**

Among 719 patients with ACI, 238 (33.1%) demonstrated BA dolichosis, including 226 (31.4%) with simple BA dolichosis and 12 (1.7%) with basilar artery dolichoectasia (BADE). Pearson correlation analyses showed that BA curve length was positively correlated with BL (*r* = 0.605). Multivariate logistic regression analysis demonstrated that current smoking (OR = 1.50, 95% CI: 1.02–2.21, *p* = 0.039), diabetes mellitus (OR = 1.66, 95% CI: 1.14–2.41, *p* = 0.008), BA diameter (OR = 3.04, 95% CI: 2.23–4.13, *p* < 0.001), BA bending (OR = 4.24, 95% CI: 2.91–6.17, *p* < 0.001) and BL (OR = 1.45, 95% CI: 1.36–1.55, *p* < 0.001) were significantly associated with BA dolichosis.

**Conclusion:**

This study suggests that BA dolichosis was common in patients with ACI, and the morphological parameters of the vertebrobasilar artery and acquired risk factors (including smoking and diabetes) were risk factors for BA dolichosis.

## 1. Introduction

Basilar artery dolichoectasia (BADE), a typical type of vertebrobasilar dolichoectasia (VBD), is characterized by elongation and dilatation of the BA. Some studies have argued that BADE may result from the combination of congenital developmental defects in the BA and the combined action of multiple risk factors for atherosclerosis (1–3) and that it may evolve dynamically (1, 4). Passero et al. (1). performed imaging follow-ups for 156 VBD patients with an average disease duration of 11.7 years and showed that 43% of patients had morphological developments in the BA, including increases in BA length (BAL), BA diameter, and bending length (BL). A large BA diameter, high bifurcation of the BA, and elongation and dilatation of the anterior cerebral artery were factors correlated with morphological developments in the BA. In addition, the morphological progression of BA may further influence the prognosis of these patients (5–7).

BADE is an uncommon vasculopathy in the Chinese Han population, and many patients with simple BA dolichosis cannot be classified as having BADE. Our previous study found that among 101 patients with acute pontine infarction, 33 patients (32.7%) presented with simple BA dolichosis, and only one patient (1.0%) developed BADE (7). Another investigation showed that in 346 community-dwelling older adults, 11 individuals (3.2%) had BA ectasia, 36 individuals (11.6%) had BA dolichosis, and only 4 individuals (1.2%) had BADE (2). However, little attention has been given to the prevalence of BA dolichosis and its associated risk factors, which is commonly observed in Chinese patients with acute cerebral infarction (ACI). The length of the BA does not remain constant (8), and its morphological remodeling may also be influenced by genetic factors (such as abnormalities in the structure and function of vascular muscle fibers and variations in different vertebrobasilar morphological indices) and acquired environmental factors (1–3). Therefore, based on this hypothesis, this study aimed to elucidate the prevalence and associated factors of BA dolichosis in the Chinese population by analyzing the clinical and imaging data of patients with ACI in our stroke unit.

## 2. Methods

### 2.1. Study design and patients

This was a cross-sectional study that purposely selected patients with ACI. All patients consecutively admitted between July 2015 and June 2019 to our department were selected according to the following inclusion criteria: age 18–80 years old, admission within 7 days after onset, and diagnosis of ACI by diffusion-weighted imaging. Patients with infarct foci involving both the anterior and posterior circulation, segmental thickening of the BA or BA aneurysms, evidence of hemodynamically severe BA stenosis (≥70%) or occlusion affecting data measurements, or incomplete clinical or imaging information were excluded. This study was approved by the Institutional Review Board of Hefei Hospital Affiliated to Anhui Medical University. Written informed consent was obtained from all patients or their guardians. All patients were registered in the Anhui Stroke Network Registry.

### 2.2. Clinical and laboratory data

Detailed clinical data were acquired from the patients, including age, sex, current smoking status, the presence of hypertension, diabetes mellitus, and dyslipidemia, as well as admission systolic and diastolic blood pressure. Hypertension was defined as a resting systolic/diastolic blood pressure of ≥140/≥90 mm Hg on repeated measurements or if the patient was taking anti-hypertension drugs. Diabetes mellitus was diagnosed when the patient had a fasting blood glucose level of ≥7.0 mmol/L, was taking oral hypoglycemic agents, or had been treated with insulin. Dyslipidemia was diagnosed if the patient had a total cholesterol level of ≥5.60 mmol/L, a triglyceride level of ≥1.81 mmol/L, or a low-density lipoprotein level of ≥3.57 mmol/L, or if the patient had taken lipid-lowering medications for these conditions. Laboratory information, including blood glucose, total cholesterol, triglycerides, low-density lipoprotein, C-reactive protein, and homocysteine levels, was systematically recorded.

### 2.3. Imaging protocol and analysis

Magnetic resonance imaging (MRI) and magnetic resonance angiography (MRA) were performed using a 1.5 Tesla MRI scanner (Siemens Healthineers, Model: Avanto I class, Germany). The geometrical parameters of the BA were analyzed by syngo 3-D VesselView. The scanning parameters and method for evaluating the BA characteristics, including BA diameter, BA curve length, BAL, BL, and BADE, were described in a previously published study (7). Among them, BAL is defined as the linear distance from the confluence point of the bilateral VAs to the initial point of the BA division into the bilateral posterior cerebral arteries, and BL is defined as the vertical distance from the bending point of the BA to the standard line of the BAL (9, 10). A BA curve length > 29.5 mm or BL > 10 mm was diagnosed as BA dolichosis, and BA ectasia was defined as a BA diameter > 4.5 mm at any location along its course. Patients meeting the above two criteria simultaneously were considered to have BADE (11). The severity of the BA bending was classified as moderate (0 < BL ≤ 10 mm) and prominent (BL > 10 mm). We further evaluated the maximum bend of the BA, which was divided into the proximal, middle, and distal portions of the BA. Image analysis was performed by two experienced neurologists, and the mean values of the above parameters were recorded as the results for further analysis. The line of the BAL was used to determine the location of BA bending (toward the right or left side or straight) (10). When there was any disagreement, a radiologist with 10 years of experience was consulted to resolve the issue. The diameters of the V4 segment of the bilateral vertebral arteries (VAs) were measured. From the vertebrobasilar artery junction, a series of three measurements with 3-mm intervals at each side was taken, and the mean diameter served as the VA diameter (12). VA dominance was defined as a difference in the diameter of both vertebral arteries of at least 0.3 mm or as an existing asymmetry in the merging of both VAs at the vertebrobasilar junction (10, 12). A Bland–Altman plot was used to analyze the agreement between the two readers.

### 2.4. Statistical analysis

All statistical analyses were performed using SPSS version 22.0 for Windows (SPSS Inc., Chicago, IL). Normally distributed variables are expressed as the mean ± standard deviation (mean ± SD), while nonnormally distributed variables are shown as the median (M) and interquartile range (IQR). Categorical variables are expressed as absolute numbers and percentages (%). Differences in continuous variables between groups were assessed by Student’s *t*-test (normally distributed) or the Mann–Whitney *U*-test (nonnormally distributed). Differences in categorical variable distributions between groups were assessed by the *χ*^2^ test or Fisher’s exact test. Univariate and multivariate logistic regression were performed to determine the factors associated with BA dolichosis. Odds ratios (ORs) and 95% confidence intervals (CIs) were subsequently calculated. Potential relationships between variables were assessed by Pearson correlation analysis, and the correlation coefficient was expressed as *r*. All tests used a two-sided *p*-value of 0.05 as a threshold for significance. All plots were drawn using GraphPad Prism software (version 8.0).

## 3. Results

### 3.1. Baseline characteristics

A total of 719 patients with ACI were included in the study, of whom 452 had anterior circulation infarction and 267 had posterior circulation infarction. The mean age was 63.7 ± 10.4 years, and 69.5% were male. Among them, 238 patients (33.1%) demonstrated BA dolichosis, including 226 patients (31.4%) with simple BA dolichosis and 12 patients (1.7%) with BADE, while other 481 patients (66.9%) had no BA dolichosis. The proportion of BA dolichosis in patients with anterior and posterior circulation infarction was 32.5% (147/452) and 34.1% (91/267), respectively, with no statistically significant difference between the two groups. Further analysis revealed that in patients aged ≤64 years (median age), the proportion of BA dolichosis in patients with anterior and posterior circulation infarction were 35.9% and 34.1%, respectively, while in patients aged >64 years, the proportion of BA dolichosis were 29.5% and 34.1%, respectively.

**Figure 1:**
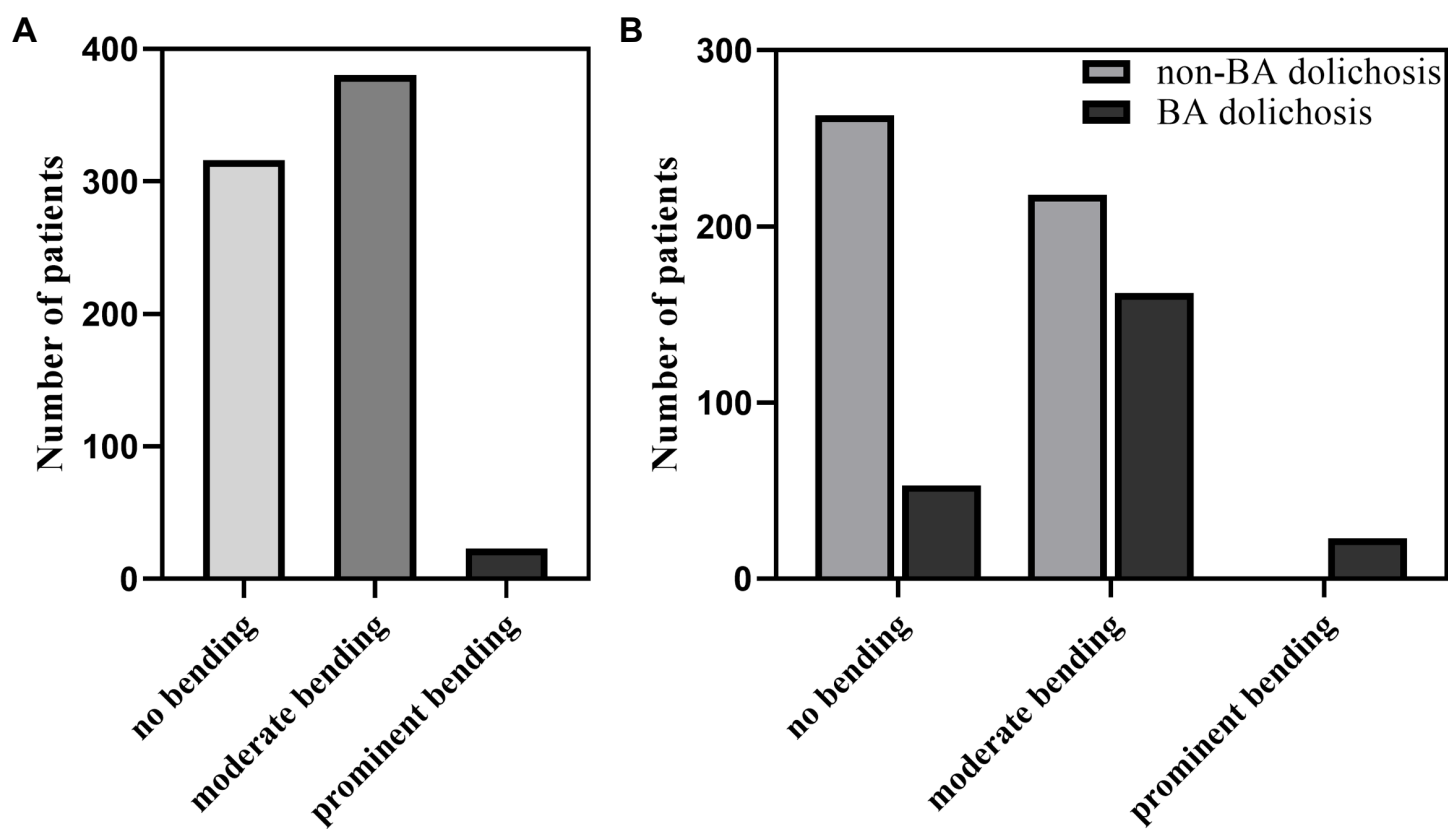
BA bending distribution in the entire study population **(A)**; BA bending distribution in patients with BA dolichosis and non-BA dolichosis **(B)**.

**Figure 2:**
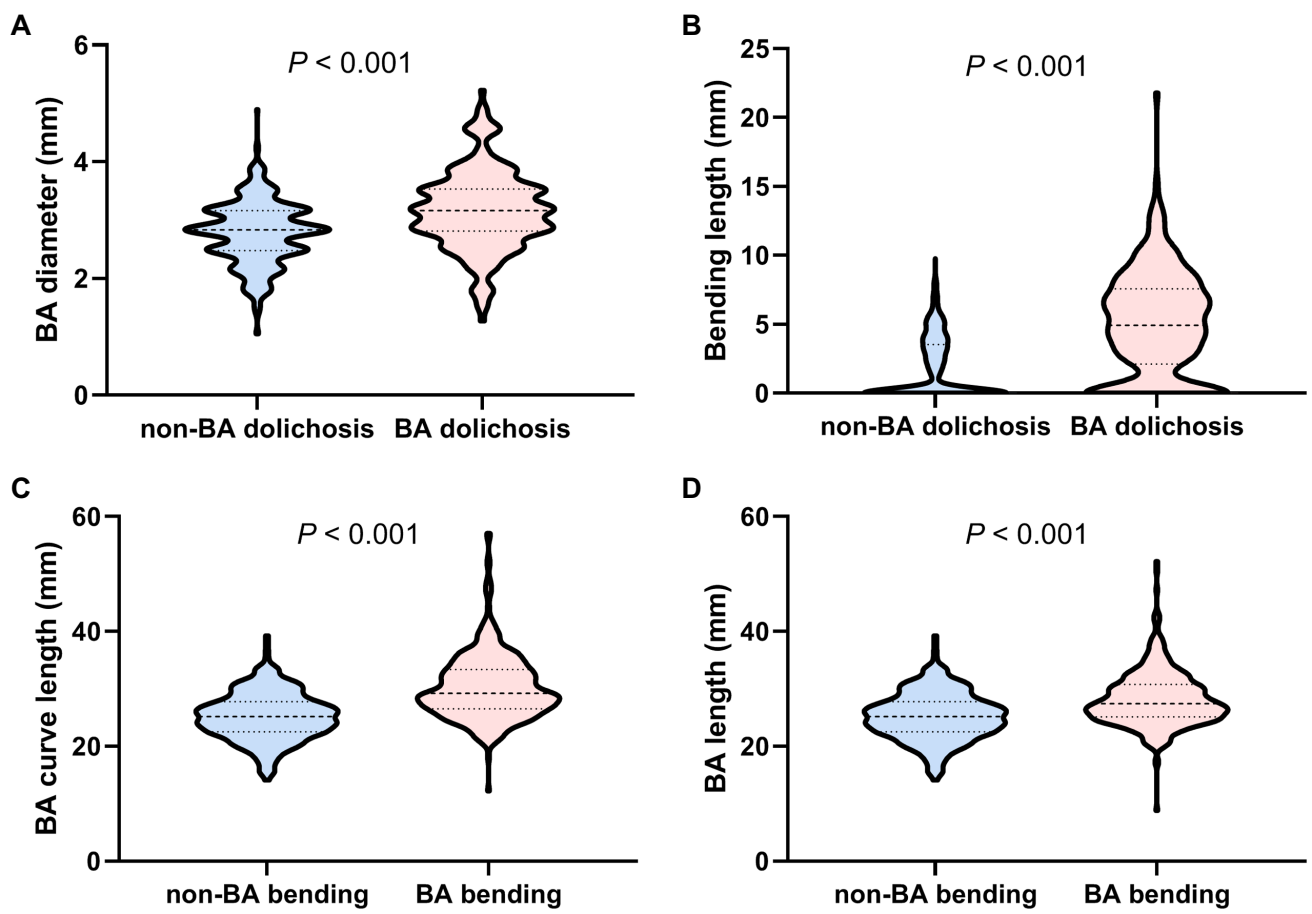
Comparison of BA diameter and BL between patients with BA dolichosis and non-BA dolichosis **(A,B)**; Comparison of BA curve length and BAL between patients with BA bending and non-BA bending **(C,D)**.

A total of 403 patients had BA bending (including 380 patients with moderate bending and 23 patients with prominent bending), and 316 patients did not have BA bending (Figure 1). BA diameter (*p* < 0.001) and BL (*p* < 0.001) were significantly higher in patients with BA dolichosis than in those with non-BA dolichosis (Figures 2A,B). The BA curve length (*p* < 0.001) and BAL (*p* < 0.001) in patients with BA bending were significantly higher than those in patients with non-BA bending (Figures 2C,D). The proportion of BA bending was significantly higher in the BA dolichosis group than in the non-BA dolichosis group (77.7% vs. 45.3%, *p* < 0.001). No patient was diagnosed with simple BA ectasia. In 378 patients (94.7%) with BA bending, the maximum bend of the BA was located at the middle of the BA, with only 9 patients bending in the proximal region of the BA and 16 patients bending in the distal portion of the BA.

### 3.2. Univariate and multivariate logistic regression analysis of factors associated with BA dolichosis

Male sex (*p* < 0.001), current smoking (*p* = 0.001), diabetes mellitus (*p* = 0.019), diastolic blood pressure (*p* = 0.013), BA diameter (*p* < 0.001), left VA diameter (*p* = 0.001), right VA diameter (*p* = 0.002), and BA bending (*p* < 0.001) were significantly greater in patients with BA dolichosis than in patients without BA dolichosis in univariate analysis (Table 1). After adjusting for variables with a potential association (variables with a *p*-value <0.1 in univariate analysis), current smoking (OR = 1.50, 95% CI: 1.02–2.21, *p* = 0.039), diabetes mellitus (OR = 1.66, 95% CI: 1.14–2.41, *p* = 0.008), BA diameter (OR = 3.04, 95% CI: 2.23–4.13, *p* < 0.001), and BA bending (OR = 4.24, 95% CI: 2.91–6.17, *p* < 0.001) were significantly associated with BA dolichosis (Table 2). When BA bending was replaced by BL, a quantitative indicator indicating the degree of BA bending, in the above logistic regression model, BL (OR = 1.45, 95% CI: 1.36–1.55, *p* < 0.001) was also significantly associated with BA dolichosis (Table 2).

**Table 1:** Univariate logistic regression analysis of factors associated with BA dolichosis in patients with ACI.

Variable	BA dolichosis group (*n* = 238)	Non-BA dolichosis group (*n* = 481)	Unadjusted OR (95%CI)	Value of *p*
**Demographic data**				
Age (years)	63.1 ± 10.3	64.0 ± 10.4	0.99 (0.98–1.01)	0.289
Male sex, *n* (%)	187 (78.6)	313 (65.1)	1.97 (1.37–2.83)	<0.001
**Risk factors**				
Current smoking, *n* (%)	86 (36.1)	119 (24.7)	1.72 (1.23–2.41)	0.002
Hypertension, *n* (%)	152 (63.9)	293 (60.9)	1.13 (0.82–1.56)	0.443
Diabetes mellitus, *n* (%)	84 (35.3)	129 (26.8)	1.49 (1.07–2.08)	0.020
Dyslipidemia, *n* (%)	72 (30.3)	124 (25.8)	1.25 (0.89–1.76)	0.205
**Blood pressure on admission**				
Systolic blood pressure (mmHg)	152.3 ± 21.1	150.1 ± 20.0	1.01 (1.00–1.01)	0.168
Diastolic blood pressure (mmHg)	90.6 ± 12.5	88.1 ± 12.1	1.02 (1.00–1.03)	0.013
**Laboratory indices**				
Blood glucose (mmol/L)	6.26 ± 2.91	6.65 ± 3.03	0.96 (0.90–1.01)	0.111
Total cholesterol (mmol/L)	4.30 ± 0.86	4.34 ± 0.98	0.95 (0.80–1.12)	0.514
Triglycerides (mmol/L)	1.99 ± 1.27	1.96 ± 1.42	1.02 (0.91–1.14)	0.776
Low-density lipoprotein (mmol/L)	2.59 ± 0.74	2.65 ± 0.86	0.91 (0.75–1.10)	0.307
C-reactive protein (mg/L)	6.57 ± 11.10	7.21 ± 13.09	1.00 (0.98–1.01)	0.511
Homocysteine (μmol/L)	16.50 ± 13.94	15.00 ± 11.17	1.01 (1.00–1.02)	0.125
**Vertebrobasilar artery features**				
BA diameter (mm)	3.17 ± 0.68	2.78 ± 0.53	3.12 (2.32–4.19)	<0.001
Left VA diameter (mm)	2.52 ± 0.94	2.29 ± 0.77	1.41 (1.16–1.72)	0.001
Right VA diameter (mm)	2.28 ± 0.81	2.09 ± 0.77	1.38 (1.13–1.70)	0.002
VA diameter difference (mm)	0.98 ± 0.93	0.85 ± 0.77	1.20 (1.00–1.44)	0.054
BL (mm)	4.97 (2.22, 7.71)	0 (0, 3.53)	1.44 (1.35–1.53)	<0.001
BA bending, *n* (%)	185 (77.7)	218 (45.3)	4.21 (2.96–6.00)	<0.001
VAD, *n* (%)	168 (70.6)	338 (70.3)	1.02 (0.72–1.14)	0.930

BA, basilar artery; VA, vertebral artery; BL, bending length; VAD, vertebral artery dominance.

**Table 2:** Multivariate logistic regression analysis of factors associated with BA dolichosis.

Models	Adjusted OR (95% CI)	Value of *p*
**Model 1 (with BA bending)**		
Current smoking	1.50 (1.02–2.21)	0.039
Diabetes mellitus	1.66 (1.14–2.41)	0.008
BA diameter	3.04 (2.23–4.13)	<0.001
BA bending	4.24 (2.91–6.17)	<0.001
**Model 2 (with BL)**		
Smoking	1.73 (1.16–2.59)	0.007
Diabetes mellitus	1.59 (1.06–2.38)	0.026
BA diameter	3.36 (2.40–4.69)	<0.001
BL	1.14 (1.36–1.55)	<0.001

### 3.3. Correlation analysis between dependent variables

**Figure 3:**
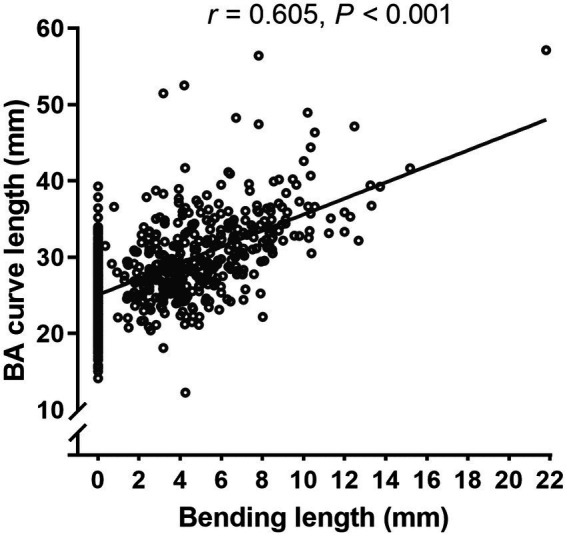
Pearson correlation analyses showed a positive correlation between BA curve length and BL (*r* = 0.605, *p* < 0.001).

Pearson correlation analyses showed a positive correlation between BA curve length and BL (*r* = 0.605, *p* < 0.001; Figure 3). In addition, BA diameter, left VA diameter, and right VA diameter were positively correlated with BA curve length, BAL, and BL, respectively, and the VA diameter difference was also significantly positively correlated with BL (Supplementary Figure).

## 4. Discussion

In the present study, we hypothesized that the length of the BA may be related to the innate geometric patterns of the vertebrobasilar artery and acquired risk factors. We found that BA dolichosis was highly associated with smoking, diabetes mellitus, BA diameter, and BA bending in patients with ACI. In addition, the BA curve length was also positively correlated with the BL, BA diameter, and VA diameter.

BADE is uncommon in stroke patients, but we found BA dolichosis to be relatively common in patients with ACI, and that there was little difference in the proportion of BA dolichosis between patients with anterior and posterior circulation infarction (32.5% vs. 34.1%). This may be related to the fact that simple BA dolichosis has a less pronounced effect on stroke than BADE, especially in young and middle-aged patients, which does not usually cause a higher proportion of posterior circulation infarction. However, this proportional difference may widen with increasing age, as suggested by the subgroup analysis of this study, which was more pronounced in patients older than 64 years. This also suggests that BA dolichosis may be influenced by congenital factors and develops when the patient reaches adulthood (1), yielding little difference in the proportion of BA dolichosis between younger patients with anterior and posterior circulation infarction, but may lead to an increased risk of posterior circulation infarction with advancing age and increasing atherosclerotic factors (13).

Although we briefly compared the BA geometrical properties between patients with and without BA dolichosis in our previous study (7), we did not evaluate the factors associated with BA dolichosis and the sample size was small. The present study had a sample size more than 7 times larger than the previous one and analyzed the factors associated with BA dolichosis more systematically. Previous studies have concluded that BADE diagnoses in healthy young people and children suggest congenital susceptibility as a potential cause of congenital developmental defects in the BA (14, 15). Pathological studies have confirmed that degeneration of the internal elastic lamina and smooth muscular atrophy are the main features of BADE in adults (16). However, it is challenging to obtain pathological data in a clinical context, but the geometric patterns of the vertebrobasilar artery that are influenced by congenital factors can be directly visualized by vascular imaging. From another perspective, this study revealed that BA curve length was positively related to BA diameter and BL; that is, a greater diameter and curvature of the BA might be related to a longer BA. The vessel radius is the most essential determinant of blood flow; a larger BA diameter leads to more blood flow and greater pulling force, thereby acting as a potential stimulus for morphological changes in the BA (e.g., elongation, ectasia, and/or curvature), especially when BA bending already exists. Multivariate analysis also demonstrated that BA diameter and BA bending were closely related to BA dolichosis, further supporting the above viewpoint. Additionally, we found that BA curve length was positively correlated with BL and that BL, a quantitative indicator representing the degree of BA bending, was another risk factor associated with BA dolichosis. However, the underlying mechanism is not clear. Presumably, in addition to being associated with congenital vascular development, the uneven blood flow within a curved BA, with the greatest blood pressure at the bend, also exacerbates the progression of BA curvature and elongation with increasing age (8).

In addition, the study by Hong et al. showed that the difference in the diameter of the VAs was the only independent predictor of moderate to severe BA dolichosis (12). Unlike their study, we only demonstrated a positive correlation between the VA diameter difference and BL. Even so, it is still generally believed that the BA usually curves in the opposite direction of the larger VA (12). The asymmetrical blood flow from the bilateral VAs might be an important hemodynamic contributor to BA mechanical changes, such as BA curvature and elongation (9, 11).

Risk factors for atherosclerosis may also play an important role in the development of BA dolichosis. The BA and VA morphological variants or structural deformation mentioned above can cause atherosclerosis, which in turn further aggravates BA dolichosis, and they can generate a vicious circle thereby increasing the risk of posterior circulation infarction (7, 13, 17, 18). Known influencing factors of BADE include aging, hypertension, diabetes mellitus, and smoking, among others, which are also targets that merit particular attention and intervention (3, 9). This study found that smoking and diabetes mellitus were also closely associated with BA dolichosis, supporting the hypothesis that atherosclerosis may be involved in the development of BA dolichosis. Previous studies have suggested that hypertension is critical in the development of VBD, especially the increase in BA diameter due to the influence of high blood pressure and subsequent hemodynamic changes (4). However, it remains unclear whether blood pressure directly affects BA length. In this study, univariate analysis revealed that the BA dolichosis group had a high proportion of hypertension and significantly elevated diastolic blood pressure, but multivariate analysis did not confirm that they were independent influencing factors of BA dolichosis. The results may need to be further verified with larger sample studies. Additionally, there was a higher proportion of males in the BA dolichosis group. Deng et al. suggested that compared with females, males had a larger BA diameter, which is associated with BA length, while the overall height of males was greater than that of females and therefore could explain the longer BA in male individuals (19).

The present study also has some limitations. First, this study only included patients with ACI. Many patients showed a high prevalence of risk factors for atherosclerosis. Therefore, a separate or comparative analysis of influencing factors of BA dolichosis in healthy people is also warranted. Second, this study did not provide a long-term follow-up of the dynamic evolution of BA curve length. Third, this study did not evaluate the flow dynamics of the vertebrobasilar artery, which may help to elucidate the mechanism of the formation and development of BA dolichosis.

## 5. Conclusion

In this study, we found that BA dolichosis was common in patients with ACI, and the morphological parameters of the vertebrobasilar artery and acquired risk factors (including smoking and diabetes) were risk factors for BA dolichosis.

## Data availability statement

The raw data supporting the conclusions of this article will be made available by the authors, without undue reservation.

## Ethics statement

The studies involving human participants were reviewed and approved by the Institutional Review Board of Hefei Hospital Affiliated to Anhui Medical University. The patients/participants provided their written informed consent to participate in this study.

## Author contributions

This work was conceptualized by MX, SC, and XW and all approved the protocol. Data collection was done by MZ, JH, PC, TG, SC, and JW. Statistical analysis was undertaken by MZ and SC. SC, MZ, and JH prepared the manuscript. MX and WX were recipients of the obtained funding and were involved in the interpretation of the data and the manuscript revision work was conceptualized by MX, SC, and WX and all approved the protocol. All authors contributed to the article and approved the submitted version.

## Funding

This study was supported by grants from Anhui Provincial Key Research and Development Plan (1804h08020233).

## Conflict of interest

The authors declare that the research was conducted in the absence of any commercial or financial relationships that could be construed as a potential conflict of interest.

## Publisher’s note

All claims expressed in this article are solely those of the authors and do not necessarily represent those of their affiliated organizations, or those of the publisher, the editors and the reviewers. Any product that may be evaluated in this article, or claim that may be made by its manufacturer, is not guaranteed or endorsed by the publisher.

## Supplementary material

The Supplementary material for this article can be found online at: https://www.frontiersin.org/articless/10.3389/fmed.2023.832878/full#supplementary-material
