# Supplementary material for: Prevalence and associated factors of basilar artery dolichosis in patients with acute cerebral infarction
Source: Front Med (Lausanne). 2023 Feb 23;10:832878. doi: 10.3389/fmed.2023.832878 (PMC9995486; doi:10.3389/fmed.2023.832878)
Supplement: Supplementary file 1 [file Data_Sheet_2.DOCX]

**Supplementary Figure legend**

**Figure.** Pearson correlation analyses showed that BA diameter, left VA diameter, and right VA diameter were positively correlated with BA curve length, BAL, and BL, respectively; the VA diameter difference was also significantly positively correlated with BL. *: *P* < 0.05.
