# Supplementary figures and images for: Prevalence and associated factors of basilar artery dolichosis in patients with acute cerebral infarction
Source: Front Med (Lausanne). 2023 Feb 23;10:832878. doi: 10.3389/fmed.2023.832878 (PMC9995486; doi:10.3389/fmed.2023.832878)

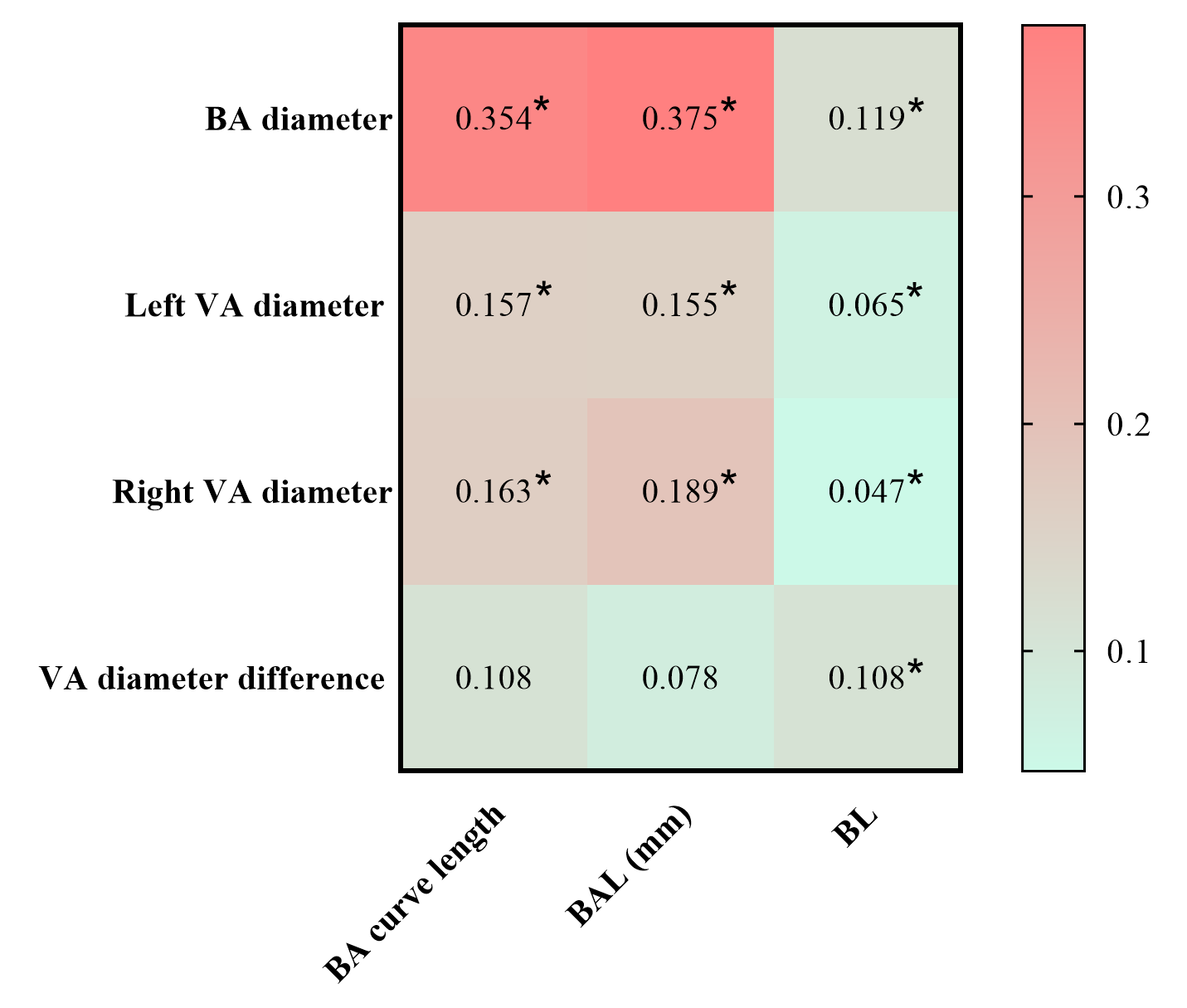

Supplement: Supplementary file 2 [file Image_1.TIF]
